# Effects of adjunctive pimavanserin and current antipsychotic treatment on QT interval prolongation in patients with schizophrenia

**DOI:** 10.3389/fpsyt.2022.892199

**Published:** 2022-09-06

**Authors:** Dragana Bugarski-Kirola, Rene Nunez, Ramzey Odetalla, I-Yuan Liu, Mary Ellen Turner

**Affiliations:** ^1^Acadia Pharmaceuticals GmbH, Basel, Switzerland; ^2^Acadia Pharmaceuticals Inc., San Diego, CA, United States

**Keywords:** pimavanserin, schizophrenia, QT interval, antipsychotics, adjunctive therapy

## Abstract

**Background:**

Pimavanserin prolongs the QT interval, with mean increases in corrected QT (QTc) of 5–8 ms, and is currently being investigated for the treatment of negative symptoms of schizophrenia.

**Objectives:**

To assess QT interval prolongation in 3 studies investigating once-daily pimavanserin as an adjunct to current antipsychotic treatment in patients with schizophrenia.

**Methods:**

Electrocardiograms were unblinded from trials in which pimavanserin or placebo was added to main antipsychotics over 6 weeks (ENHANCE), 26 weeks (ADVANCE), and up to 78 weeks (ongoing 52-week, open-label extension study [study 035]) of treatment. Antipsychotic treatment was permitted throughout these studies. The 3 most frequently used antipsychotic treatments were examined—aripiprazole (including long-acting injectable), risperidone (including long-acting injectable), and olanzapine. QT intervals were corrected (QTc) using Fridericia's method, with elevated risk defined as either postbaseline value maximum of >500 ms or change from baseline to postbaseline maximum of >60 ms.

**Results:**

Of patients treated with adjunctive pimavanserin in ENHANCE, there were no postbaseline QTc values >481 ms; one patient in each of the risperidone and aripiprazole groups had change from baseline to postbaseline maximum >60 ms. More patients had change from baseline to postbaseline maximum ranging from 31 to 60 ms in the risperidone plus adjunctive placebo group (*n* = 5; 6.6%) than those in the risperidone plus adjunctive pimavanserin group (*n* = 3, 4.1%). In the pimavanserin plus antipsychotic group of ADVANCE, one patient had postbaseline QTc value >481 ms, and one patient treated with aripiprazole had change from baseline to postbaseline maximum of >60 ms. In study 035, a change from double-blind baseline to overall postbaseline maximum >60 ms occurred in one patient treated with aripiprazole and pimavanserin and in one patient treated with risperidone and pimavanserin. Similar proportions of patients had changes from double-blind baseline to post double-blind baseline maximum between 31 and 60 ms across treatments. No adverse events associated with an increase in the QTc interval were reported.

**Conclusions:**

Adjunctive pimavanserin with background antipsychotic treatment showed no evidence of QTc prolongation >500 ms postbaseline, consistent with previously reports on QT prolongation with pimavanserin.

## Introduction

Augmentation with a second antipsychotic drug is a common strategy for treating patients with schizophrenia who do not exhibit a complete response to initial antipsychotic therapy (1, 2). However, treatment with multiple antipsychotic drugs has been associated with increased burden of adverse events (1). In particular, patients with schizophrenia who are receiving antipsychotic polypharmacy may be at a higher risk for QT interval prolongation—which is associated with an increased risk of mortality (3)—than patients who do not receive multiple antipsychotic drugs (4, 5). Antipsychotic drugs as a group have long been known to have the potential to cause prolongation of the QT interval (5, 6), which has been attributed to blockade of the cardiac potassium channel human ether-a-go-go-related gene (7, 8). For example, quetiapine, ziprasidone, and iloperidone include warnings in their prescribing information that caution against use with other QT prolonging medications or in patients at risk for QT prolongation (9–11). Several additional risk factors unrelated to pharmacotherapy, namely, low potassium or magnesium levels, marked bradycardia, poorly controlled advanced heart failure and inherited long QT syndrome, could potentially lead to arrhythmias or torsade de pointes, especially when combined with the QT effects of antipsychotic drugs (6). When these well-known risk factors are absent, clinically significant QT/ QT interval corrected for heart rate (QTc) prolongations occur infrequently among patients receiving atypical antipsychotic drugs (12).

Pimavanserin is a selective 5-HT_2A_ receptor inverse agonist/antagonist approved by the United States Food and Drug Administration for the treatment of hallucinations and delusions associated with Parkinson's disease psychosis (13). Pimavanserin is currently being investigated for its potential to treat negative symptoms of schizophrenia. During early clinical development of pimavanserin, a thorough QTc study was conducted in healthy adults receiving pimavanserin 17 mg (*n* = 57) or 68 mg (*n* = 68), and the results showed that the effect of pimavanserin on the QTc interval was small (17 mg: maximal mean change [90% upper CI], 4.7 ms [6.8 ms]; 68 mg: 13.9 ms [15.9 ms]) (14, 15). Furthermore, in the pivotal studies leading to pimavanserin approval in Parkinson's disease psychosis, the maximum mean change in QTc interval in patients receiving pimavanserin 34 mg was 6.9 ms (upper CI, 10.0 ms); one patient developed a QTc >500 ms (16). However, consideration of patients developing QT prolongation was implemented in future pimavanserin study protocols to mitigate this risk. Two completed studies, ENHANCE and ADVANCE, have investigated pimavanserin treatment in patients with schizophrenia receiving a background antipsychotic drug (17, 18). In both studies, the use of background antipsychotic drugs was limited to those without a warning for QT interval prolongation in their prescribing information. Although these therapies are not without risk of QT interval prolongation, they likely have a lower risk than those with a warning. In addition, qualifying patients from these studies could participate in study 035, a 52-week ongoing open-label extension study. In the 6-week ENHANCE study, pimavanserin vs. placebo added to background antipsychotic treatment resulted in a nonsignificant positive trend in reduction of Positive and Negative Syndrome Scale total score and Clinical Global Impression-Severity (CGI-S) score. Safety and tolerability were comparable with those of placebo (17). In ADVANCE, pimavanserin vs. placebo added to background antipsychotic treatment resulted in significantly greater improvement of negative symptoms as assessed with the 16-item Negative Symptom Assessment total score during the course of 26 weeks. Pimavanserin was generally safe and well tolerated (18).

An increase in QT interval prolongation is included in a warning in the prescribing information for the 34-mg dose of pimavanserin approved for hallucinations and delusions associated with Parkinson's disease psychosis, along with a precaution to avoid use with drugs that also increase the QT interval or in patients with risk factors for prolonged QT interval (13). However, in the overall population of ENHANCE, values >480 ms were not seen for any patient's QT interval corrected by Fridericia's method (QTcF) (17). Change from baseline in QTcF >60 ms was reported in 2 patients (1.1%) in the pimavanserin group and no patients in the placebo group (17). In ADVANCE, no patients had QTcF values >500 ms, and a change from baseline in QTcF >60 ms was reported in one patient (0.5%) in the pimavanserin group and in none in the placebo group (18). Furthermore, in both ENHANCE and ADVANCE, there were very low rates of serious treatment-emergent adverse events (TEAEs) and discontinuation due to TEAEs. In ENHANCE, for pimavanserin vs. the placebo group, the rates of serious TEAEs (1 vs. 1%, respectively) and discontinuation for TEAE (2.5 vs. 0%, respectively) were both below 3%. In addition, in ADVANCE, for pimavanserin vs. the placebo group, the rates of serious TEAEs (2 vs. <1%, respectively) and discontinuation for TEAE (5 vs. 3%, respectively) were both ≤ 5%. Both studies included assessment and encouragement of adherence to background antipsychotic drugs (aripiprazole [including long-acting injectable], olanzapine, risperidone [including long-acting injectable]) and pimavanserin as part of their study designs and achieved adherence rates of ≥90% (19), making results from these studies suitable for continued evaluation of using pimavanserin and a background antipsychotic drug together. This analysis used data from ENHANCE, ADVANCE and the associated 52-week ongoing open-label extension study to evaluate QT interval prolongation in patients with schizophrenia treated with their background antipsychotic drug and once-daily pimavanserin.

## Methods

### Study design

Data from 3 studies were analyzed to determine the effects of adjunctive pimavanserin on QT interval prolongation in patients with schizophrenia taking select antipsychotic drugs (Table 1). ENHANCE (NCT02970292) was a 6-week, randomized, double-blind, placebo-controlled phase 3 study in patients with schizophrenia and inadequate response to their current antipsychotic treatment (17). ADVANCE (NCT02970305) was a 26-week, randomized, double-blind, placebo-controlled phase 2 study in patients with schizophrenia and predominant negative symptoms while on treatment with an antipsychotic medication (18). Study 035 (NCT03121586) is an ongoing 52-week, ongoing open-label extension study of patients from ENHANCE and ADVANCE (database extraction date, April 28, 2021).

**Table 1:** Included studies.

**Study**	**Study design**	**Treatment**	**Population**	**N**	**Time of QTcF assessments**
ENHANCE (ACP-103-034; NCT02970292)	Phase 3, randomized, double-blind, placebo-controlled study	Pimavanserin or placebo (1:1) for 6 weeks	Adults aged ≥50 years with schizophrenia and an inadequate response to current antipsychotic treatment	396 •198 pimavanserin •198 placebo	Screening, baseline and weeks 4 and 6 (EOS/ET)
ADVANCE (ACP-103-038; NCT02970305)	Phase 2, randomized, double-blind, placebo-controlled study	Pimavanserin or placebo (1:1) for 26 weeks	Adults aged ≥50 years with negative symptoms of schizophrenia	403 •201 pimavanserin •202 placebo	Screening, baseline, and weeks 14 and 26 (EOS/ET)
Study 035 (ACP-103-035; NCT03121586)	Ongoing open-label extension study of patients from ENHANCE and ADVANCE	Pimavanserin for 52 weeks	Extension of ENHANCE and ADVANCE	648 •315 double-blind pimavanserin •333 double-blind placebo	Open-label baseline and weeks 2, 28, and 52 (EOS/ET)

EOS, end of study; ET, early termination; QTcF, QT interval using Fridericia's correction method.

Study designs and primary results of the ENHANCE and ADVANCE studies have been reported previously (17, 18). Both studies included screening periods of up to 4 weeks, a treatment period of 6 or 26 weeks, respectively, and 4-week follow-up for patients who did not enter the extension study or discontinued treatment early. In the ENHANCE and ADVANCE studies, a flexible dosing design for pimavanserin treatment was used. The starting dose of pimavanserin 20 mg once daily for the first 2 weeks could be adjusted to 10 mg or 34 mg once daily based on investigator's assessment of clinical response. No dose adjustments were allowed after week 3 (ENHANCE) or week 8 (ADVANCE) visits. In study 035, patients who completed the ENHANCE week 6 visit or the ADVANCE week 26 visit, met all eligibility criteria, and would benefit from continued treatment (investigator's discretion) were started on 20 mg once daily for the first 2 weeks before investigator's assessment of dose adjustment and remained on their assigned dose for up to 52 weeks. No changes in the dose of ongoing antipsychotic were allowed throughout the duration of the study. Single 12-lead echocardiogram (ECG) recordings were collected at baseline (ENHANCE and ADVANCE) and at weeks 4 and 6 (ENHANCE) or weeks 14 and 26 (ADVANCE).

### Patients

#### Inclusion criteria

Men and women aged 18–55 years and diagnosed with schizophrenia (*Diagnostic and Statistical Manual of Mental Disorders, Fifth Edition* criteria) (20) at least 1 year before randomization were enrolled. Patients were medically stable for at least 12 weeks prior to screening and had a screening and baseline score of at least 4 on the CGI-S (indicating overall schizophrenia severity for ENHANCE) or Clinical Global Impression of Schizophrenia Scale-Severity (indicating severity of negative symptoms of schizophrenia in ADVANCE).

Patients were receiving ongoing treatment with one of the following antipsychotic drugs at doses that were in line with local prescribing information: aripiprazole (including long-acting injectable), asenapine, brexpiprazole, cariprazine, lurasidone, olanzapine, or risperidone (including long-acting injectable). Before screening, patients had no change in oral antipsychotic dose for 4 weeks or no change in long-acting injectable antipsychotic drug for 16 weeks. A stable dose of antipsychotic drug was required throughout the screening period and throughout the study, including the 52-week ongoing open-label extension study.

#### Exclusion criteria

Patients with a comorbid psychiatric disorder other than schizophrenia based on the Structured Clinical Interview for DSM-5, Clinical Trial Version (SCID-5-CT), family or personal history of symptoms of long QT syndrome, a QRS interval <120 ms and QTcF ≥460 ms or a QRS interval ≥120 ms and QTcF ≥480 ms at screening or baseline, and/or treatment with a medication that prolongs the QT interval were excluded from this analysis.

All patients provided signed informed consent. All patients had a designated caregiver who supported compliance with study protocols.

### Statistical analysis

The safety analysis sets from ENHANCE, ADVANCE, and study 035 used for these analyses included patients who were randomized and received at least one dose of the study drug. ECG tracings were evaluated by a central laboratory, and QTc was summarized by treatment group. The QTcF was categorized by time (≤450, 451–480, 481–500 and >500 ms), and change from baseline (≤10, 11–30, 31–60 and >60 ms). QTcF and change from baseline in QTcF were considered potentially clinically important at >500 and >60 ms, respectively.

In this *post hoc* analysis, ECG data were analyzed in subgroups defined by the most frequent background antipsychotic treatment: aripiprazole (including long-acting injectable), risperidone (including long-acting injectable) or olanzapine. ECG data from a “no risperidone” subgroup were analyzed to understand the effects of antipsychotic drugs in patients with low or moderate risk of QTc prolongation, given that the main metabolite of risperidone (9-hydroxy paliperidone) has a QTc prolongation warning in its prescribing information (21). This “no risperidone” group included patients who were treated with aripiprazole, asenapine, brexpiprazole, cariprazine, lurasidone, or olanzapine.

## Results

This analysis included 396 patients (198, pimavanserin; 198, placebo) who were randomized in ENHANCE. Overall, the mean age was 37.1 years (standard deviation [SD], 0.5 years), and the last dose level of pimavanserin was 10 mg in 2.5% of patients (5 of 198), 20 mg in 44.4% (88 of 198), and 34 mg in 53.0% (105 of 198). In ADVANCE, 403 randomized patients (201, pimavanserin; 202, placebo) were included. Overall, the mean age was 37.2 years (SD, 0.5 years), and the last dose level of pimavanserin was 10 mg in 1.5% of patients (3 of 201), 20 mg in 45.3% (91 of 201), and 34 mg in 53.2% (107 of 201). Of those patients who completed the ENHANCE or ADVANCE studies, 648 patients completed study 035.

### ENHANCE study

**Figure 1:**
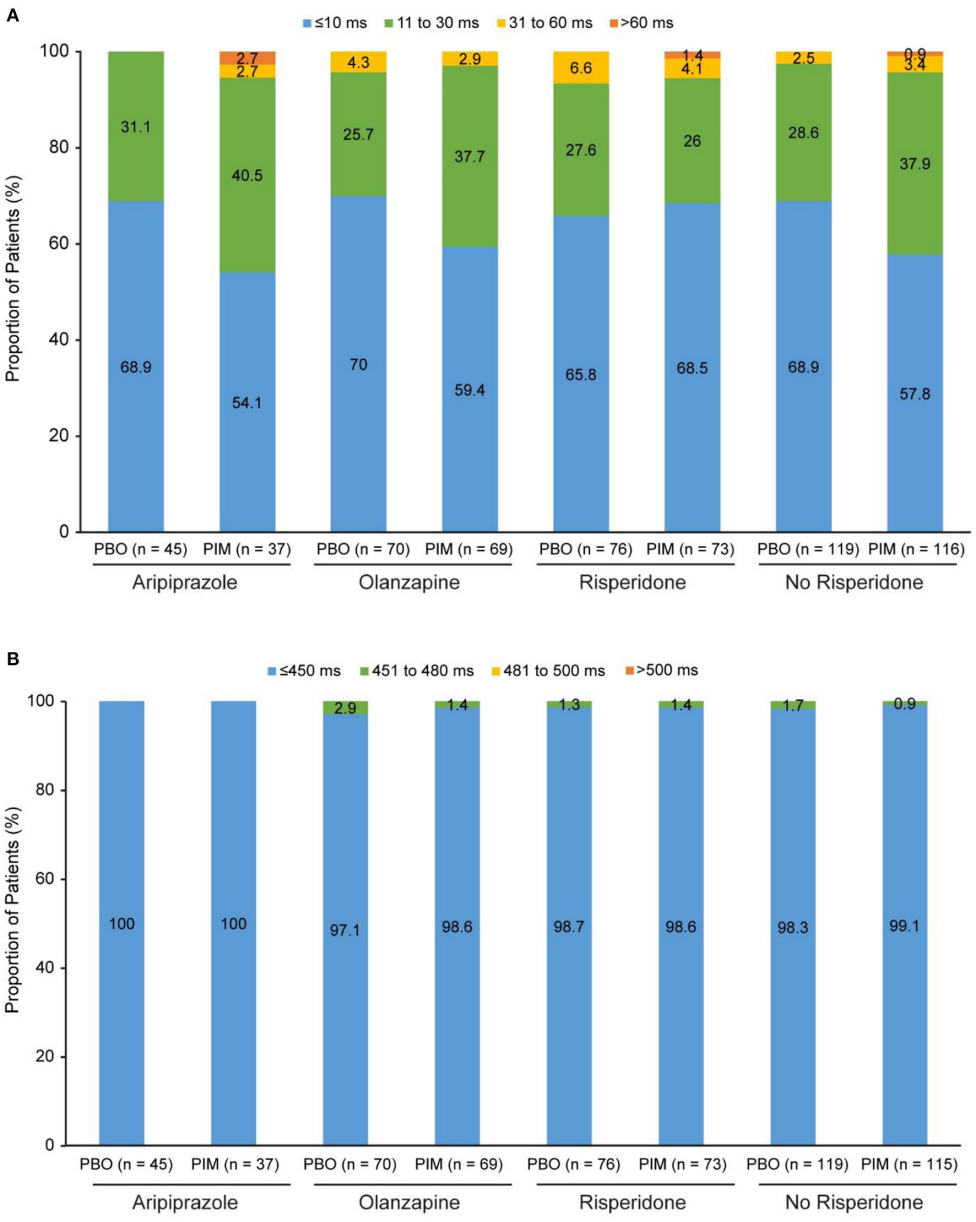
ENHANCE: **(A)** Change in QTcF from baseline to overall postbaseline maximum and **(B)** QTcF overall postbaseline maximum. PBO, placebo; PIM, pimavanserin; QTcF, QT interval using Fridericia's correction method.

In total, 78 of patients receiving pimavanserin and 78 of patients receiving placebo were treated with risperidone, 71 and 71 with olanzapine, and 38 and 45 with aripiprazole at baseline, respectively. In addition to the most frequent background antipsychotic treatments, the number of patients receiving pimavanserin and placebo and treated with other antipsychotics were as follows: asenapine (1 and 0, respectively), brexpiprazole (4 and 1), cariprazine (1 and 1), and lurasidone (5 and 2). Mean QTcF intervals at baseline were generally similar across antipsychotic drug subgroups (Table 2). Mean change in QTcF from baseline to week 6 ranged from −1.1 to −0.2 ms for patients receiving placebo and 0.1 to 2.4 ms for patients receiving pimavanserin (Table 2). For change from baseline to overall postbaseline maximum, there were no patients in the placebo subgroups and one patient in each of the risperidone 4 mg and aripiprazole 400 mg with pimavanserin subgroups with a change >60 ms (Figure 1A). More patients had a change from baseline to overall postbaseline maximum ranging from 31 to 60 ms with placebo (5 of 76; 6.6%) than in the risperidone with adjunctive pimavanserin group (3 of 73; 4.1%). There were no postbaseline QTcF values >480 ms in the placebo or pimavanserin subgroups (Figure 1B; Table 2).

**Table 2:** ENHANCE: QTcF at baseline and on-study.

	**Aripiprazole**		**Olanzapine**		**Risperidone**		**No risperidone** ^**a**^	
	**PBO** **(*N =* 45)**	**PIM** **(*N =* 38)**	**PBO (*N =* 71)**	**PIM (*N =* 71)**	**PBO (*N =* 78)**	**PIM (*N =* 78)**	**PBO** **(*N =* 120)**	**PIM (*N =* 120)**
Baseline QTcF, *n^*b*^*	45	38	71	71	78	78	120	120
Mean (SE), ms	389.5 (2.97)	398.2 (3.48)	399.5 (2.05)	401.0 (2.11)	401.3 (2.05)	400.2 (2.37)	395.1 (1.73)	399.8 (1.69)
Change from baseline to week 6, *n^*b*^*	42	35	68	64	72	67	114	107
Mean (SE), ms	−0.8 (1.62)	0.1 (2.61)	−0.3 (1.85)	2.4 (1.89)	−1.1 (1.94)	0.1 (2.12)	−0.2 (1.26)	2.0 (1.50)
Overall postbaseline maximum, *n*^c^	45	37	70	69	76	73	119	116
≤ 450 ms, *n* (%)	45 (100)	37 (100)	68 (97.1)	68 (98.6)	75 (98.7)	72 (98.6)	117 (98.3)	115 (99.1)
451–480 ms, *n* (%)	0 (0.0)	0 (0.0)	2 (2.9)	1 (1.4)	1 (1.3)	1 (1.4)	2 (1.7)	1 (0.9)
481–500 ms, *n* (%)	0 (0.0)	0 (0.0)	0 (0.0)	0 (0.0)	0 (0.0)	0 (0.0)	0 (0.0)	0 (0.0)
>500 ms, *n* (%)	0 (0.0)	0 (0.0)	0 (0.0)	0 (0.0)	0 (0.0)	0 (0.0)	0 (0.0)	0 (0.0)
Change from baseline to overall postbaseline maximum, *n*^c^	45	37	70	69	76	73	119	116
≤ 10 ms, *n* (%)	31 (68.9)	20 (54.1)	49 (70.0)	41 (59.4)	50 (65.8)	50 (68.5)	82 (68.9)	67 (57.8)
11–30 ms, *n* (%)	14 (31.1)	15 (40.5)	18 (25.7)	26 (37.7)	21 (27.6)	19 (26.0)	34 (28.6)	44 (37.9)
31–60 ms, *n* (%)	0 (0.0)	1 (2.7)	3 (4.3)	2 (2.9)	5 (6.6)	3 (4.1)	3 (2.5)	4 (3.4)
>60 ms, *n* (%)	0 (0.0)	1 (2.7)	0 (0.0)	0 (0.0)	0 (0.0)	1 (1.4)	0 (0.0)	1 (0.9)

^a“^No risperidone” group included patients who were treated with aripiprazole, asenapine, brexpiprazole, cariprazine, lurasidone or olanzapine.

^b^Number of patients with non-missing values at the given visit and treatment group.

^c^Number of patients with a least one postbaseline value for the given treatment group, used as denominator for percentages shown in categories below.

N, patients randomized to a given treatment; PBO, placebo; PIM, pimavanserin; QTcF, QT interval using Fridericia's correction method; SE, standard error.

### ADVANCE study

**Figure 2:**
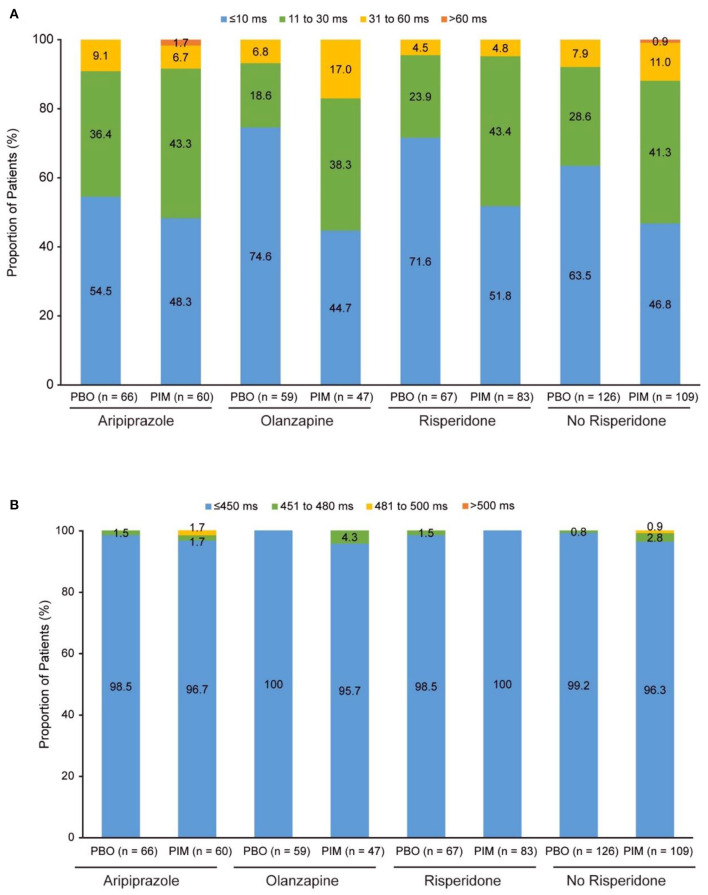
ADVANCE: **(A)** Change in QTcF from baseline to overall postbaseline maximum and **(B)** QTcF overall postbaseline maximum. PBO, placebo; PIM, pimavanserin; QTcF, QT interval using Fridericia's correction method.

In total, 86 patients receiving pimavanserin and 69 patients receiving placebo were treated with risperidone, 63 and 69 with aripiprazole, and 50 and 62 with olanzapine at baseline, respectively. In addition to the most frequent background antipsychotic treatments, the number of patients receiving pimavanserin and placebo and treated with other antipsychotics were as follows: asenapine (0 and 0, respectively), brexpiprazole (0 and 0), cariprazine (0 and 0), and lurasidone (2 and 2). Mean change from baseline to week 26 ranged from −1.6 to 3.2 ms for patients receiving placebo and 3.0–6.4 ms for patients receiving pimavanserin (Table 3). More patients in the “no risperidone” with placebo and pimavanserin subgroups had a change from baseline to postbaseline maximum of 31 to 60 ms (22 of 235; 9.4%) than patients treated with risperidone (7 of 150; 4.7%) (Figure 2A). A change from baseline to postbaseline maximum >60 ms was reported in one patient treated with aripiprazole 15 mg and pimavanserin (Figure 2A). There were no patients with overall postbaseline maximum >500 ms, and an overall postbaseline maximum >480 ms was reported in one patient who received treatment with pimavanserin in the aripiprazole 15 mg subgroup (Figure 2B; Table 3).

**Table 3:** ADVANCE: QTcF at baseline and on-study.

	**Aripiprazole**		**Olanzapine**		**Risperidone**		**No risperidone** ^**a**^	
	**PBO** **(*N =* 69)**	**PIM** **(*N =* 63)**	**PBO** **(*N =* 62)**	**PIM** **(*N =* 50)**	**PBO** **(*N =* 69)**	**PIM** **(*N =* 86)**	**PBO** **(*N =* 133)**	**PIM** **(*N =* 115)**
Baseline QTcF, *n^*b*^*	69	63	62	50	69	86	133	115
Mean (SE), ms	396.0 (2.65)	397.3 (2.20)	399.5 (2.51)	398.6 (2.51)	401.8 (2.03)	400.3 (1.82)	398.0 (1.82)	398.0 (1.62)
Change from baseline to week 26, *n^*b*^*	56	54	54	41	63	78	110	97
Mean (SE), ms	3.2 (2.27)	6.4 (2.46)	−1.4 (1.90)	5.1 (3.30)	−1.6 (2.08)	3.0 (1.79)	1.0 (1.49)	5.7 (1.99)
Overall postbaseline maximum, *n*^c^	66	60	59	47	67	83	126	109
≤ 450 ms, *n* (%)	65 (98.5)	58 (96.7)	59 (100)	45 (95.7)	66 (98.5)	83 (100)	125 (99.2)	105 (96.3)
451–480 ms, *n* (%)	1 (1.5)	1 (1.7)	0 (0.0)	2 (4.3)	1 (1.5)	0 (0.0)	1 (0.8)	3 (2.8)
481–500 ms, *n* (%)	0 (0.0)	1 (1.7)	0 (0.0)	0 (0.0)	0 (0.0)	0 (0.0)	0 (0.0)	1 (0.9)
>500 ms, *n* (%)	0 (0.0)	0 (0.0)	0 (0.0)	0 (0.0)	0 (0.0)	0 (0.0)	0 (0.0)	0 (0.0)
Change from baseline to overall postbaseline maximum, *n*^c^	66	60	59	47	67	83	126	109
≤ 10 ms, *n* (%)	36 (54.5)	29 (48.3)	44 (74.6)	21 (44.7)	48 (71.6)	43 (51.8)	80 (63.5)	51 (46.8)
11–30 ms, *n* (%)	24 (36.4)	26 (43.3)	11 (18.6)	18 (38.3)	16 (23.9)	36 (43.4)	36 (28.6)	45 (41.3)
31–60 ms, *n* (%)	6 (9.1)	4 (6.7)	4 (6.8)	8 (17.0)	3 (4.5)	4 (4.8)	10 (7.9)	12 (11.0)
>60 ms, *n* (%)	0 (0.0)	1 (1.7)	0 (0.0)	0 (0.0)	0 (0.0)	0 (0.0)	0 (0.0)	1 (0.9)

^a^“No risperidone” group included patients who were treated with aripiprazole, asenapine, brexpiprazole, cariprazine, lurasidone or olanzapine.

^b^Number of patients with non-missing values at the given visit and treatment group.

^c^Number of patients with a least one postbaseline value for the given treatment group, used as denominator for percentages shown in categories below.

N, patients randomized to a given treatment; PBO, placebo; PIM, pimavanserin; QTcF, QT interval using Fridericia's correction method; SE, standard error.

### Study 035

In this analysis of data from study 035, 171 patients who completed either the ENHANCE or ADVANCE study were treated with aripiprazole, 214 with olanzapine, and 253 with risperidone at baseline. In addition to the most frequent background antipsychotic treatments, the number of patients receiving pimavanserin and treated with other antipsychotics at baseline were as follows: 1 patient who completed either the ENHANCE or ADVANCE study were treated with asenapine, 1 with brexpiprazole, 1 with cariprazine, and 7 with lurasidone. These patients received either placebo or pimavanserin in the double-blind studies, but all patients received pimavanserin during the open-label portion of study 035.

**Figure 3:**
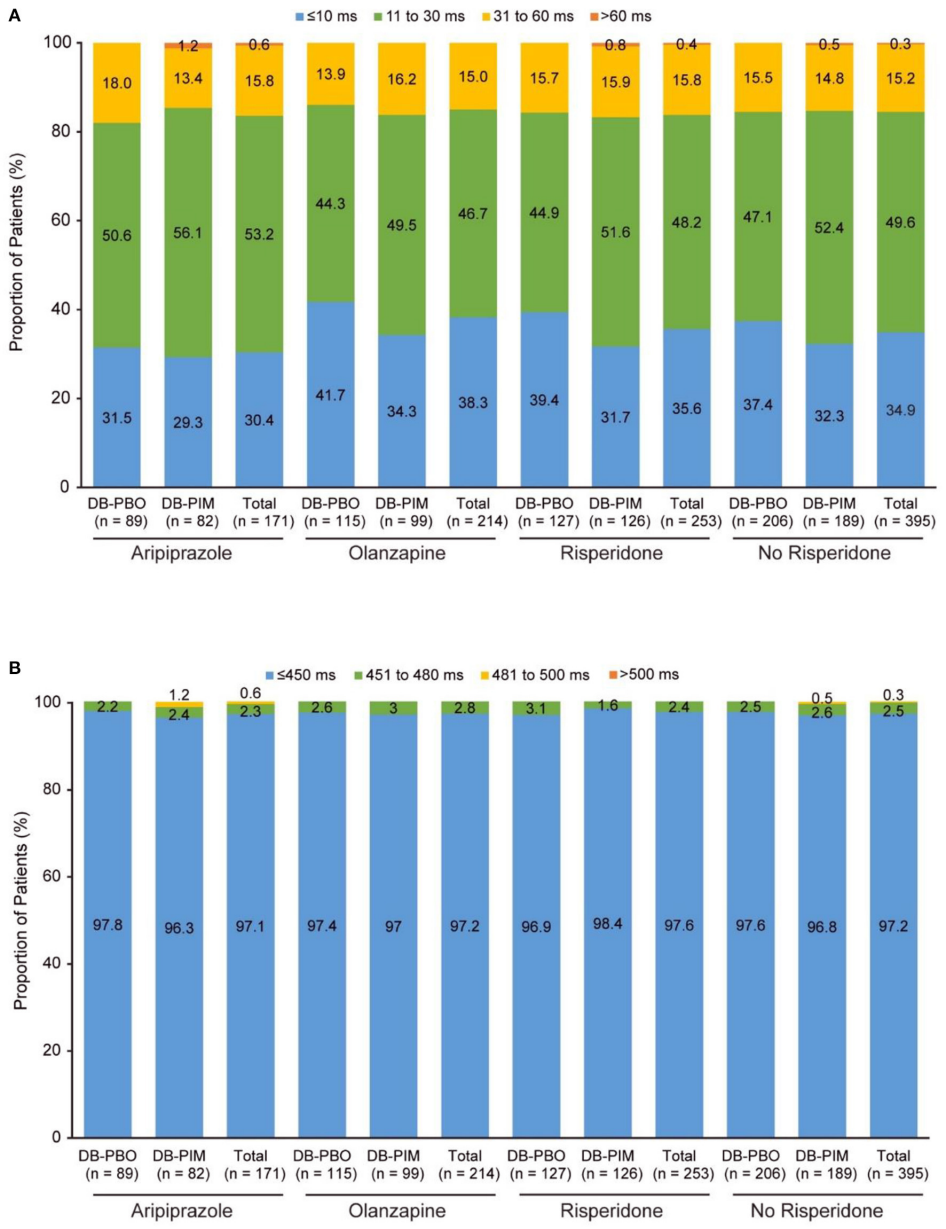
Study 035^a^: **(A)** Change in QTcF from double-blind baseline to overall postbaseline maximum and **(B)** QTcF overall postbaseline maximum. ^a^After the double-blind studies (ENHANCE and ADVANCE), patients could enroll in study 035, an extension study. Patients are grouped by their randomized treatment (placebo or pimavanserin) from the ENHANCE and ADVANCE trial, but all patients were treated with pimavanserin throughout the 52-week extension study. DB, double-blind; PBO, placebo; PIM, pimavanserin; QTcF, QT interval using Fridericia's correction method.

At the time of the interim analysis, 130 patients treated with aripiprazole, 172 with olanzapine and 197 with risperidone had non-missing values. Mean changes from double-blind baseline to open-label week 52 QTcF were similar across subgroups (2.5–5.7 ms; Table 4). A change from double-blind baseline to overall postbaseline maximum >60 ms occurred in one patient treated with aripiprazole 15 mg QD and pimavanserin and one patient treated with risperidone 4 mg BID and pimavanserin (Figure 3A). Similar proportions of patients had changes from double-blind baseline to post double-blind baseline maximum 31 to 60 ms across treatments (Figure 3A). A post double-blind baseline maximum of 481 to 500 ms was observed in one patient treated with aripiprazole 15 mg QD (Figure 3B; Table 4).

**Table 4:** Study 035: QTcF at double-blind baseline and on-study.

	**Aripiprazole**			**Olanzapine**			**Risperidone**			**No risperidone** ^**a**^		
	**DB PBO OL PIM** **(*N =* 89)**	**DB PIM OL PIM ** **(*N =* 82)**	**Total ** **(*N =* 171)**	**DB PBO OL PIM ** **(*N =* 115)**	**DB PIM OL PIM ** **(*N =* 99)**	**Total ** **(*N =* 214)**	**DB PBO OL PIM ** **(*N =* 127)**	**DB PIM OL PIM ** **(*N =* 126)**	**Total ** **(*N =* 253)**	**DB PBO OL PIM ** **(*N =* 206)**	**DB PIM OL PIM ** **(*N =* 189)**	**Total ** **(*N =* 395)**
DB baseline QTcF, *n^*b*^*	89	82	171	115	99	214	127	126	253	206	189	395
Mean (SE), ms	393.7 (2.11)	398.6 (2.02)	396.0 (1.47)	399.0 (1.78)	402.0 (1.72)	400.4 (1.24)	400.8 (1.53)	399.9 (1.75)	400.4 (1.16)	396.5 (1.36)	400.3 (1.27)	398.3 (0.94)
Change from DB baseline to OL week 52, *n^*b*^*	68	62	130	91	81	172	97	100	197	160	148	308
Mean (SE), ms	3.8 (2.02)	5.3 (2.54)	(1.60)	2.5 (1.77)	5.0 (2.01)	3.7 (1.33)	5.7 (1.74)	3.4 (1.80)	4.5 (1.25)	3.1 (1.32)	5.0 (1.53)	4.0 (1.01)
Overall postbaseline maximum, *n*^c^	89	82	171	115	99	214	127	126	253	206	189	395
≤ 450 ms, *n* (%)	87 (97.8)	79 (96.3)	166 (97.1)	112 (97.4)	96 (97.0)	208 (97.2)	123 (96.9)	124 (98.4)	247 (97.6)	201 (97.6)	183 (96.8)	384 (97.2)
451–480 ms, *n* (%)	2 (2.2)	2 (2.4)	4 (2.3)	3 (2.6)	3 (3.0)	6 (2.8)	4 (3.1)	2 (1.6)	6 (2.4)	5 (2.5)	5 (2.6)	10 (2.5)
481–500 ms, *n* (%)	0 (0.0)	1 (1.2)	1 (0.6)	0 (0.0)	0 (0.0)	0 (0.0)	0 (0.0)	0 (0.0)	0 (0.0)	0 (0.0)	1 (0.5)	1 (0.3)
>500 ms, *n* (%)	0 (0.0)	0 (0.0)	0 (0.0)	0 (0.0)	0 (0.0)	0 (0.0)	0 (0.0)	0 (0.0)	0 (0.0)	0 (0.0)	0 (0.0)	0 (0.0)
Change from DB baseline to overall post-DB baseline maximum, *n*^c^	89	82	171	115	99	214	127	126	253	206	189	395
≤ 10 ms, *n* (%)	28 (31.5)	24 (29.3)	52 (30.4)	48 (41.7)	34 (34.3)	82 (38.3)	50 (39.4)	40 (31.7)	90 (35.6)	77 (37.4)	61 (32.3)	138 (34.9)
11–30 ms, *n* (%)	45 (50.6)	46 (56.1)	91 (53.2)	51 (44.3)	49 (49.5)	100 (46.7)	57 (44.9)	65 (51.6)	122 (48.2)	97 (47.1)	99 (52.4)	196 (49.6)
31–60 ms, *n* (%)	16 (18.0)	11 (13.4)	27 (15.8)	16 (13.9)	16 (16.2)	32 (15.0)	20 (15.7)	20 (15.9)	40 (15.8)	32 (15.5)	28 (14.8)	60 (15.2)
>60 ms, *n* (%)	0 (0.0)	1 (1.2)	1 (0.6)	0 (0.0)	0 (0.0)	0 (0.0)	0 (0.0)	1 (0.8)	1 (0.4)	0 (0.0)	1 (0.5)	1 (0.3)

^a^No risperidone group included patients who were treated with aripiprazole, asenapine, brexpiprazole, cariprazine, lurasidone or olanzapine.

^b^Number of patients with non-missing values at the given visit and cohort.

^c^Number of patients with a least one postbaseline value for the given cohort, used as denominator for percentages shown in categories below.

N, OL patients for the given cohort; DB, double-blind; OL, open-label; PBO, placebo; PIM, pimavanserin; QTcF, QT interval using Fridericia's correction method; SE, standard error.

### Cardiac-related adverse events

There were no clinically meaningful changes in ECG parameters for ENHANCE or ADVANCE (17, 18). No patients in ENHANCE or ADVANCE experienced serious treatment-emergent adverse events (TEAEs) related to cardiac disorders. In ENHANCE, 2 of 198 placebo-treated patients (0.7%) and 3 of 198 pimavanserin-treated patients (1.5%) reported cardiac disorders as a TEAE. One pimavanserin-treated patient (0.5%) had a related TEAE of cardiac disorder (palpitations) that was the only cardiac-related cause for treatment discontinuation; however, this patient regularly consumed energy drinks that may have contributed to this TEAE. In ADVANCE, 3 of 202 placebo-treated patients (1.5%) and 3 of 201 pimavanserin-treated patients (1.5%) reported cardiac disorders as a TEAE. Two pimavanserin-treated patients (1.0%) had a cardiac disorder as a related TEAE; for both patients, a mild occurrence of tachycardia was reported. No patients had a cardiac-related cause of treatment discontinuation.

## Discussion

In this *post hoc* analysis assessing QT interval prolongation in patients with schizophrenia who received pimavanserin added to background antipsychotic treatment in 3 large studies, the mean changes in QTc prolongation were minimal and generally similar to those of the placebo treatment arms. There was no evidence of postbaseline QTc prolongation >500 ms in patients treated with adjunctive pimavanserin and either aripiprazole, olanzapine, or risperidone. QTcF change >60 ms was observed in few patients in the pimavanserin subgroups.

Results of a meta-analysis show that antipsychotic drugs can increase the QTc interval (5) and highlight the need to consider assessing separately each and every combination of therapies (ie, clozapine with risperidone vs. clozapine with ziprasidone) because of the differential effects that may occur on QTc interval. Furthermore, consideration of cardiovascular safety of medications used to treat schizophrenia are particularly relevant, as a cohort study conducted in the United States found that individuals with schizophrenia were at an increased risk of premature death, and that death from cardiovascular disease was 3.6 times more likely than in the general population (22). Additionally, drug-induced QT prolongation is associated with a higher risk of all-cause and cardiovascular mortality (23). Because pimavanserin also causes QTc prolongation, combining antipsychotic drugs with pimavanserin could compound treatment-induced QTc interval prolongation to cause aberrant cardiovascular events that may lead to mortality. QT interval prolongation in patients who received individual background antipsychotic drugs (i.e., asenapine, brexpiprazole, cariprazine, or lurasidone) with adjunctive pimavanserin remains unknown because patients receiving other antipsychotic drugs either were not recruited or because there were only a few cases. Few studies have been designed to investigate the effect that polypharmacy, especially with multiple antipsychotic drugs, has on QT prolongation (4). Therefore, it is important to understand the effect of the combination of antipsychotic therapy with other drug interventions on the QTc interval to treat schizophrenia effectively while minimizing the risk of cardiac events.

The results of this *post hoc* analysis indicate that pimavanserin 34 mg, known to prolong QT interval by ~5–8 ms (13), does not produce further clinically relevant QT interval prolongation when used as an adjunctive treatment with any of 3 commonly used antipsychotic drugs (i.e., risperidone, olanzapine, or aripirazole) in patients with schizophrenia. Furthermore, although ADVANCE and ENHANCE were randomized, double-blind studies of patients who had not previously received treatment with pimavanserin, study 035 was an open-label extension of these studies, in which all patients had received pimavanserin treatment. This study provided long-term data on pimavanserin exposure. Of note, because the ENHANCE study targeted patients with inadequate response to antipsychotic drugs, a significant portion of enrolled patients were receiving high doses of antipsychotic drugs allowed per prescribing information specifications; 4.8% of patients received a risperidone dose of >8 mg (17). Use of higher doses may increase the risk for QTc prolongation (23). However, in both ENHANCE and ADVANCE, no safety concerns related to an increase in the QTc interval were reported, and no clinically relevant changes were observed in ECG parameters (17, 18). Furthermore, as noted above, the incidence of TEAEs and extrapyramidal symptoms were similar between pimavanserin and placebo in both ENHANCE and ADVANCE, indicating pimavanserin was well-tolerated. Together, these results show that under the conditions of these controlled trials, combining antipsychotic and pimavanserin therapy has a nominal effect on QTc interval. Nevertheless, routine monitoring of ECG parameters is still warranted for proper disease management of cardiac safety.

Although it is understood that antipsychotics can cause QTc prolongation with varying degrees of intensity (ie, adjunctive treatment of pimavanserin with aripiprazole possibly having a lower risk for QTc prolongation than pimavanserin with risperidone), our study demonstrated adjunctive treatment with risperidone and aripiprazole lead to similar QTc prolongations. To address these findings, we reviewed individual patient profiles of patients with adjunctive treatment of pimavanserin and aripiprazole or risperidone. For this analysis, we included patients with QTc prolongation of >60 ms and QTc >450 ms. We found that in ADVANCE, of the 4 patients with QTc prolongation from baseline in the aripiprazole group, all were females with three being aged 53–55 and two with medical comorbidities such as diabetes mellitus and hypertension as well as concomitant medications that may affect the QTc such as escitalopram. These findings support the notion that additional risk factors such as sex, prior treatments, and comorbidities may lead to infrequent cases of QTc prolongation despite the fact that the adjunctive treatment of pimavanserin with aripiprazole is known to have a lower risk for QTc prolongation than pimavanserin with risperidone.

One potential challenge of examining the safety of antipsychotic drugs, especially in combination, is that the nonadherence is frequently observed among populations of patients with schizophrenia (24, 25). Thus, a lack of safety effects could reflect either the safety of treatment or insufficient exposure to one or more of the medications. In the ENHANCE and ADVANCE studies, pharmacokinetic assessments were used to screen patients to ensure that they were adherent to their background antipsychotic drug, and similar assessments throughout the study along with compulsory caregiver participation encouraged adherence to both pimavanserin and the background antipsychotic drug (19). With these study design measures, adherence was maintained throughout the studies. Even during long-term treatment over 26 weeks in ADVANCE, >80% of patients were adherent to both pimavanserin and background antipsychotic drug at all timepoints. The high levels of adherence allowed reliable interpretation of the results of these studies.

### Limitations

The results of this analysis must be interpreted in the context of limitations inherent to the study designs, which were not developed specifically to examine changes in QTc interval. It was not possible to conduct a pooled analysis of these data because of differences in designs and durations of these 3 studies. Therefore, the results of each study have been presented separately and analyzed using descriptive statistics. Furthermore, not all patients received the maximum dose of pimavanserin in these studies (34 mg) because of the flexible dosing regimen. As patients receiving other concomitant medications with potential significant risk of QTc prolongation were excluded from these studies, these results may not be generalizable to patients receiving other medications. Additionally, only selected antipsychotic drugs that did not have a warning of QTc prolongation in their prescribing information were permitted as background antipsychotic drugs; therefore, the effect of pimavanserin and other atypical antipsychotic drugs associated with QTc prolongation remains unknown.

## Conclusions

In these studies, findings were consistent with those of previous reports of QTc prolongation with pimavanserin used as a monotherapy. Thus, adjunctive pimavanserin added to ongoing antipsychotic treatment did not result in further increase in QTc prolongation. No safety concerns related to an increase in the QTc interval were reported. In conclusion, pimavanserin added to ongoing antipsychotic treatment with aripiprazole, olanzapine, or risperidone had a minimal effect on QTc prolongation, and changes in QTc prolongation were generally similar to those seen with placebo.

## Data availability statement

The original contributions presented in the study are included in the article/supplementary material, further enquiries can be directed to the corresponding author/s. Given the ongoing phase 3 studies in negative symptoms of schizophrenia, the research data collected for the study, including individual participant data, will not be made available on request at this time because it is anticipated that these data may be part of future regulatory submissions.

## Ethics statement

Ethical approval was not provided for this study on human participants because this is a pooled analysis; however, for each study included, the Investigator or designee provided the Institutional Review Board/Ethics Committee with all requisite materials, including a copy of the protocol, informed consent forms, and any subject information or advertising materials. The study was not initiated until the IRB/EC provided written approval of the protocol and the ICFs and until approved documents had been obtained by the Investigator and copies received by the Sponsor. The patients/participants provided their written informed consent to participate in this study.

## Author contributions

All authors have made substantial contributions to the design and interpretation of the work, drafted or revised the work for important intellectual content, approved the final version to be published and agreed to be accountable for all aspects of the work in ensuring that questions related to the accuracy or integrity of any part of the work are appropriately investigated and resolved.

## Conflict of interest

This study was funded by Acadia Pharmaceuticals Inc. The funder was involved in medical writing and editorial support. Authors DB-K, RN, RO, I-YL, and MT are employees of and hold stock and stock options in Acadia Pharmaceuticals Inc.

## Publisher's note

All claims expressed in this article are solely those of the authors and do not necessarily represent those of their affiliated organizations, or those of the publisher, the editors and the reviewers. Any product that may be evaluated in this article, or claim that may be made by its manufacturer, is not guaranteed or endorsed by the publisher.
